# Exposure to HT-2 toxin causes oxidative stress induced apoptosis/autophagy in porcine oocytes

**DOI:** 10.1038/srep33904

**Published:** 2016-09-23

**Authors:** Yue Zhang, Jun Han, Cheng-Cheng Zhu, Feng Tang, Xiang-Shun Cui, Nam-Hyung Kim, Shao-Chen Sun

**Affiliations:** 1 College of Animal Science and Technology, Nanjing Agricultural University, Nanjing 210095, China; 2 Department of Animal Sciences, Chungbuk National University, Cheongju 361-763, Korea

## Abstract

T-2 toxin is a main type A trichothecene mycotoxin which is the most toxic trichothecence. T-2 toxin has posed various toxic effects on human and animals in vigorous cell proliferation tissues like lymphoid, hematopoietic and gastrointestinal tissues, while HT-2 toxin is the major metabolite which is deacetylated by T-2 toxin. In this study, we focused on the toxic effects of HT-2 on porcine oocyte maturation. We treated the porcine oocyte with HT-2 toxin *in vitro*, and we first found that HT-2 treatment inhibited porcine oocyte polar body extrusion and cumulus cell expansion. We observed the disrupted meiotic spindle morphology after treatment, which might be due to the reduced p-MAPK protein level. Actin distribution was also disturbed, indicating that HT-2 affects cytoskeleton of porcine oocytes. We next explored the causes for the failure of oocyte maturation after HT-2 treatment. We found that HT-2 treated oocytes showed the increased ROS level, which indicated that oxidative stress had occurred. We also detected autophagy as well as early apoptosis in the treatment oocytes. Due to the fact that oxidative stress could induced apoptosis, our results indicated that HT-2 toxin caused oxidative stress induced apoptosis and autophagy, which further affected porcine oocyte maturation.

T-2 toxin is one of the type A trichothecene mycotoxins that are produced by different *Fusarium* species (mainly *Fusarium sporptrichiodes*, *Fusarium poae* and *Fusarium langsethide*) that infect crops in the fields or during storage^1^, which is the most cytotoxic member of the trichothecene family^2^. T-2 toxin represents a contaminant of considerable concern to human and animal health. At extremely high doses, it can cause shock-like syndrome that can result in death^3^. Deacetylation of T-2 toxin results in HT-2 toxin, which was the major metabolite of T-2 toxin^4^. T-2 toxin and HT-2 toxin are often found together. Concerning the toxicity, T-2 and HT-2 toxin exhibit similar toxic properties^5^. As a kind of trichothecenes, HT-2 toxin is supposed to be having multiple inhibitory effects on eukaryote cells including inhibition of protein, DNA and RNA synthesis, mitochondrial function, toxic effects on cell division and membrane, and inducer of apoptosis and a programmed cell death response^6^.

T-2 toxin is a potent inhibitor of the eukaryotic protein synthesis in different cell lines, and T-2 toxin also inhibits DNA and RNA from synthesizing^7^. Several studies showed that it also inhibits the mitochondrial electron transport system, mitochondrial function, mitochondrial protein synthesis, augmented lipid peroxidation^5^,^8^. Cell membrane disruption and toxic effect of cell proliferation and cell division were found in several cell lines with T-2 toxin exposure^1^,^9^. Multiorgan effects including emesis, diarrhea, weight loss, nervous disorders, cardiovascular alteration, immune suppression, hemostatic derangements, skin toxicity and bone marrow damage are also caused by T-2 toxin^10^.

Several studies have shown that oxidative stress could mediate the cytotoxicity of T-2 toxin^11^,^12^. T-2 toxin could also induce apoptosis in lymphoid and hematopoietic tissues, intestinal crypt epithelial cells, ovarian granulosa cells^13^,^14^,^15^,^16^. It is shown that T-2 toxin exposure induces apoptosis through oxidative stress in rat ovarian granulosa cells^17^. A study showed that Zearalenone, another toxin of mycotoxin, induces a high level of autophagy in Leydig cells^18^. T-2 toxin was also shown to affect reproductive system. Experiments have shown that oral exposure to low dose T-2 toxin could significantly retard the follicle maturation and ovulation^19^. T-2 toxin has been reported to readily pass through the placenta and can be distributed to fetal tissues in rats^20^, resulting in the induction of embryo/fetal death, fetal brain damage and fetal skeletal malformation^21^. However, there is still no reports for the toxic effects of T-2 or HT-2 toxins on porcine oocytes. For reproductive system, oocyte maturation is one key process. Fully-grown oocytes are arrested at the germinal vesicle breakdown (GV) stage in mature ovarian follicles and only after being released from the follicle, they resume meiosis. After germinal vesicle breakdown (GVBD), the oocytes then enter metaphase I (MI), followed by peripheral spindle migration and first polar body extrusion. After this, the oocytes enter metaphase II (MII) and stay at this stage until fertilization. Actin and spindle have significant roles in mammalian oocyte maturation. After GVBD, actin surrounds the GV and promotes chromosome congression^22^. After spindle formation, the meiotic spindle migrates and anchors onto the cortex in an actin-dependent manner^23^,^24^. Then actin, as well as myosin, facilitate the formation of a contractile ring and promote first polar body extrusion^25^.

Although T-2 toxin has adverse effects on various organs and tissues, its effects and regulatory mechanisms on the maturation of porcine oocytes is remained unknown. The objective of this study was to investigate the effects of HT-2 on the maturation of porcine oocyte. We detected cytoskeletal dynamics, oxidative stress, early apoptosis and autophagy of HT-2 treated porcine oocytes. And the results showed that altered ROS level mediated apoptosis and autophagy might be the reasons for the failure of porcine oocyte maturation after HT-2 treatment.

## Results

### HT-2 toxin exposure affects the polar body extrusion and cumulus expansion in porcine oocytes

**Figure 1:**
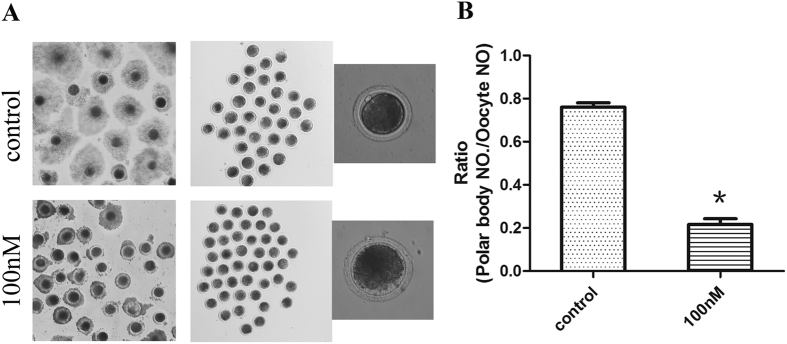
HT-2 toxin effects on the maturation rate of porcine oocytes. (**A**) GV (germinal vesicle) oocytes were cultured for 44 hour. Cumulus cell expansion occurred in control group, while after exposure to HT-2 toxin, cumulus cell expansion failed. A large proportion of oocytes developed to MII stage in the control group, showing with a polar body; while most of oocytes in treatment group failed to develop to the MII stage with no polar body. (**B**) The rate of oocyte polar body extrusion after HT-2 treatment, indicating that the oocytes maturation decreased significantly after exposure to HT-2 toxin. At least three independent experiments and more than 30 oocytes were examined in each experimental group. *p < 0.05.

To investigate the possible effects of HT-2 toxin on the maturation of porcine oocytes, we cultured porcine oocytes with different concentrations of HT-2 toxin, including 10 nM, 50 nM, 100 nM, and the average maturation rate were 73.75 ± 3.87% n = 193, 45.81 ± 2.10% n = 160, 21.67 ± 2.62% n = 181 respectively compared with the control group (76.04 ± 2.04%, n = 189), and we chose 100 nM as final concentration. The oocytes were cultured for 44 hours, and the extrusion of the first polar body was defined as nuclear maturation. The cumulus expansion did not occur in the 100 nM-treated group while in control group cumulus cells expanded well (Fig. 1A). We then examined the ratio of polar body extrusion in control and HT-2 treated groups and the rate of polar body extrusion was significantly reduced when compared with control group (21.67 ± 2.62% vs 76.04 ± 2.04%; p < 0.05; Fig. 1B). The results suggest that exposure to HT-2 toxin induces the failure of polar body extrusion in porcine oocytes.

### HT-2 effects on spindle morphology and actin distribution in porcine oocytes

**Figure 2:**
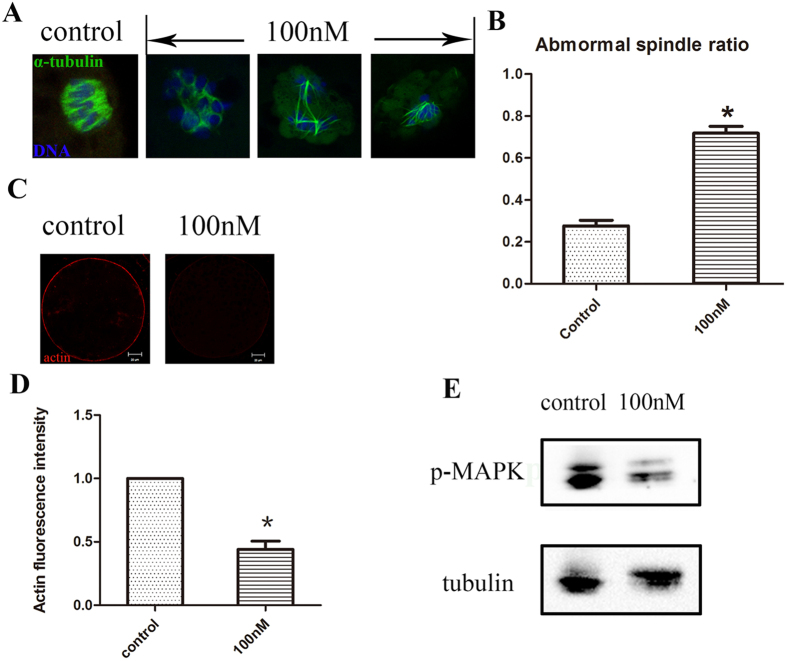
HT-2 toxin effects on spindle morphology and actin expression. (**A**) Spindle morphologies were disrupted after exposure to HT-2 toxin. In the control group, the oocyte showed normal spindle morphology, while in the treatment group the spindle formation failed, showing with no poles spindle or bad shape spindle. α-tubulin, green; DNA, blue. (**B**) Abnormal spindle rate was increased after exposure to HT-2 toxin than the control group. (**C**) Actin expression in the control and HT-2 toxin treatment groups. Actin expression was reduced after exposure to HT-2 toxin. Actin, red; DNA, blue. (**D**) Statistical comparisons of membrane actin fluorescence intensity for control and HT-2 toxin treatment groups. Actin fluorescence intensities in the membrane were reduced in treatment group. (**E**) Cropped blots are used in this figure, and the gels have been run under the same experimental conditions. Full-length blots are included for key data in the supplementary information. Western blot results showed that p-MAPK level was significantly reduced after exposure to HT-2 toxin. *p < 0.05. Bar = 20 μm.

In order to find the possible reason for the failure of first polar body extrusion, we then examined actin distribution and spindle formation. We examined the oocytes at metaphase I in control group and HT-2 treated group. As shown in Fig. 2A, most oocytes exhibit normal spindle morphology and chromosome alignment, while in HT-2 treated group spindle formation were disrupted, disorganized spindles or multipolar spindles were observed. The proportion of abnormal spindles in the HT-2 treated group was significantly higher than that in the control group (71.77 ± 2.33% n = 134 vs 27.6 ± 1.91%, n = 123; p < 0.05; Fig. 2B).

We also examined the actin filaments. For oocytes in HT-2 treated group, actin fluorescence intensities at the plasma membrane were significantly lower than those in control group (Fig. 2C). Statistical analysis showed that the actin distribution levels at the plasma membrane in HT-2 treated group were significantly decreased in comparison with control group (0.44 ± 0.07 vs 1.0; p < 0.05; Fig. 2D).

We then examined the p-MAPK expression level, which is related with microtubule organization. Western blot result showed that p-MAPK level was significantly reduced after exposure to HT-2 toxin (Fig. 2E). Of all these results, we could speculate that HT-2 toxin exposure induced disrupted meiotic spindle formation and actin distribution.

### HT-2 treatment results in oxidative stress in porcine oocytes

**Figure 3:**
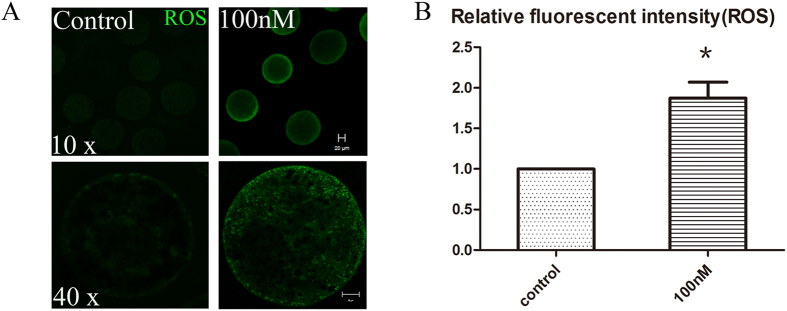
HT-2 toxin exposure causes oxidative stress in porcine oocytes. (**A**) Oocytes were stained with DCHFDA to detect intracellular reactive oxygen species (ROS) levels at the MI stage. ROS level was significantly increased after exposure to HT-2 toxin. Bar = 20 μm. (**B**) Average ROS fluorescence intensity in porcine oocytes. The ROS fluorescence intensity was increased significantly after HT-2 treatment. *p < 0.05. Bar = 20 μm.

To investigate whether oxidative stress was induced by HT-2 exposure in porcine oocyte, we examined the intracellular reactive oxygen species (ROS) levels. After oocytes in both control and treatment group reached MI stage, they were collected to determine the ROS levels. The entire cytoplasm of HT-2 exposure oocytes exhibited stronger DCFH-DA fluorescence intensity than those in control group (Fig. 3A). Statistical analysis indicated that ROS levels were significantly increased after exposure to HT-2 toxin (1.87 ± 0.20 vs 1.0; p < 0.05; Fig. 3B).

### HT-2 toxin causes early-apoptosis and autophagy in porcine oocytes

**Figure 4:**
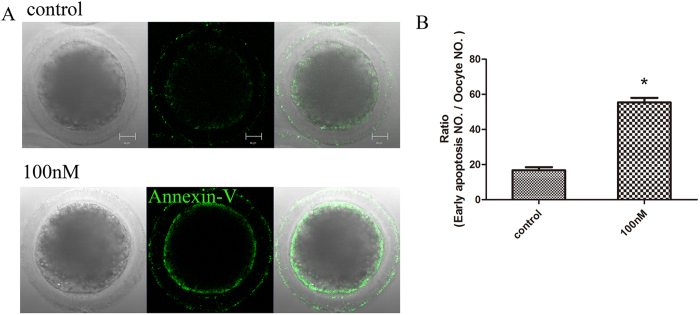
Early apoptosis was occurred among MI stage after HT-2 toxin exposure in porcine oocytes. (**A**) Oocytes were stained with a FITC-conjugated Annexin-V. In control oocytes there were no fluorescent signals on the zona pellucida, whereas in HT-2 treated porcine oocytes, early apoptosis occurred and fluorescent signals were observed on the membrane. Annexin-V, green. (**B**) Percentages of oocytes exhibiting early apoptosis. The rate of oocytes with early apoptosis after HT-2 treatment was significantly increased compared with the control group. *p < 0.05. Bar = 20 μm.

As T-2 toxin induces apoptosis in several cell lines, we then examined whether apoptosis was occurred in HT-2 treated oocytes by using Annexin V staining. As shown in Fig. 4A, early apoptosis signal arose in treatment group with strong green fluorescent signals observed on cytomembrane. In control group, the signals were with slight fluorescent or only observed on the zona pellucida. Statistical analysis indicated that the ratio of porcine oocytes with early apoptosis in HT-2 treated group is significantly higher than that in control group (55.41 ± 2.58%, n = 126 vs 16.80 ± 1.68%, n = 118; p < 0.05; Fig. 4B).

**Figure 5:**
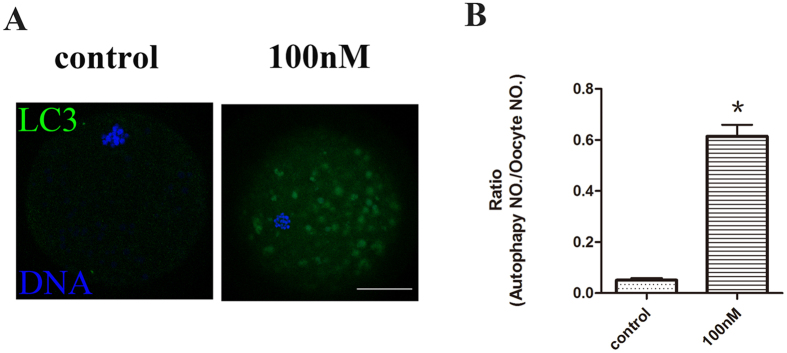
HT-2 exposure induces porcine oocytes autophagy. (**A**) Confocal laser scanning microscopic images of autophagy organization in porcine oocytes by LC3 antibody staining. Green LC3 signal dots were observed in many of the HT-2 treated porcine oocytes, which indicated autophagy occurred in treatment group. LC3, green; DNA, blue. Bar = 20 μm. (**B**) Percentages of oocytes exhibiting autophagy. *p < 0.05.

To investigate whether HT-2 toxin induces autophagy in porcine oocytes, we examined the LC3 level by immunofluorescent staining. Our result showed that LC3 fluorescent level was increased in treatment group, there were several LC3 signal dots among the cytoplasm of the treated oocytes (Fig. 5A). The percentage of oocytes that exhibited autophagy was significantly higher in the HT-2 treated group (61.43 ± 4.53%, n = 135 vs 5.12 ± 0.58%, n = 141; p < 0.05; Fig. 5B).

## Discussion

Finding out the causes/mechanism of how HT-2 induces detrimental effects on oocytes is important to avoid the adverse effects. In this study, we investigated HT-2 effects on porcine oocytes by assessing meiotic spindle formation, actin distribution, oxidative stress, autophagy and early apoptosis. Our results indicated that exposure to HT-2 toxin had adverse effects on meiotic spindle formation, actin distribution, oxidative stress, apoptosis and autophagy, which may underlie the reduced quality of oocytes maturation.

Several studies have shown that mycotoxins had influence embryonic development *in vitro* and oocytes maturation. There were evidences indicated that zearalenone (ZEN), as well as deoxynivalenol (DON), has negative effects on embryonic development of zygotes and on meiotic progression of porcine oocytes^26^. Our previous work also showed that zearalenone and deoxynivalenol affected the maturation of mouse and porcine oocytes. In present study, we assumed that HT-2 toxin has adverse effects on porcine oocyte maturation.

To confirm our hypothesis, we first found that after incubation, the maturation rate of porcine oocytes was decreased in HT-2-treated group. To investigate the reason of reduced oocytes maturation rate, we examined the porcine oocyte actin filaments and microtubules. Actin filaments regulate meiotic spindle movements and initiate cytokinesis for small polar body extrusion, while microtubules form the meiotic spindle that drives chromosome congression and segregation^23^. In our previous studies, ZEN and DON disrupted meiotic spindle formation^27^,^28^.Our results showed that a large proportion of HT-2 treated oocytes had aberrant meiotic spindle morphologies, and the plasma membrane actin distributions were reduced. To investigate the reason of abnormal spindle assembly we examined p-MAPK level, and we found that p-MAPK protein level was reduced. As MAPK participated in the regulation of microtubule organization and meiotic spindle assembly, we assumed that the changed p-MAPK level was the reason of disrupted spindle assembly. To summary this part of results, we showed that HT-2 toxin affected porcine oocyte maturation, which was confirmed by the disrupted actin filaments distribution and spindle formation.

We then tried to explore the causes of this toxic effect of HT-2. Oxidative damage caused by T-2 toxin was considered as one of the underlying mechanisms for T-2 toxin induced cytotoxicity, DNA damage, and apoptosis^29^. Either the overproduction of reactive oxygen species (ROS) or decrease of cellular antioxidant levels may induce oxidative stress^30^. Studies showed that ROS are key signaling molecules in various physiological processes, including meiotic resumption, oocyte development and maturation, follicular atresia, corpus luteum function and luteolysis^31^,^32^. Mammalian oocytes and embryos are extremely sensitive to ROS^33^. Our results showed that HT-2 exposure induced excessive ROS generation in porcine oocytes, indicating the oxidative stress occurs, which indicate the conserved roles for HT-2 on oxidative stress, which was one reason for the failure of oocyte maturation.

As mentioned above, T-2 toxin is known to induce oxidative stress, and oxidative stress is one of the underlying mechanisms of apoptosis, we then examined early apoptosis in HT-2 treated porcine oocytes. Several studies suggested that T-2 toxin induced apoptosis was regulated by ROS-mediated mitochondrial pathway in various cell lines^16^,^34^. Our results were in accordance with the previous studies and early apoptosis occurred in HT-2 treated porcine oocytes. It has been shown that ROS play an essential role in the activation of autophagy, and in turn, autophagy serves to reduce oxidative damage^35^. Atg 5, a autophagy gene, could mediate apoptosis. Since oxidative stress and early apoptosis were detected in our study, we then decided to examine autophagy as an index. It was reported that autophagy affects maternal mRNA degradation and apoptosis of porcine parthenotes developing *in vitro*^36^. While in low concentration of rapamycin, a chemical autophagy inducer, promotes enhancement of the nuclear and cytoplasmic maturation of porcine oocytes^37^. Moreover, autophagy affects apoptosis in mouse embryos^38^. Therefore, we supposed that autophagy may have effects on maturation of porcine oocytes. Then we detected autophagy level of oocytes treated with HT-2 toxin. Our results showed that autophagy occurred in oocytes exposure to HT-2 toxin and has negative effects on maturation of oocytes, which indicated that HT-2 induced autophagy affected porcine oocyte maturation rate.

In conclusion, our results suggest that HT-2 toxin might have toxic effects on cytoskeletal integrity, and induced oxidative stress, mediated apoptosis and autophagy in porcine oocytes. These altering might be the underlying reasons of the reduced quality of oocytes exposed to HT-2 toxin.

## Materials and Methods

### Oocytes harvesting and *in vitro* maturation

The experiments were conducted in accordance with the Animal Research institute Committee guidelines of Nanjing Agricultural University, China. This study was approved by the Committee of Animal Research Institute, Nanjing Agricultural University, China. The ovaries were obtained from prepubertal gilts at a local slaughterhouse and transported to our laboratory within 2 hours in 0.9% physiological saline and was maintained at 35 °C in a thermos bottle. The ovaries were washed with sterile saline and stored at 37 °C. The cumulus-oocyte complexes (COCs) were aspirated from 2–8 mm antral follicles by using a 10 ml disposable syringe with an 18 G needle. COCs contained in the sediment were washed in the DPBS and the oocytes with an intact and compact cumulus were selected. COCs were washed three times with maturation medium before maturation culture. The medium was an improved TCM-199 (Sigma, St. Louis, MO, USA) supplemented with 75 μg/ml of penicillin, 50 μg /ml of streptomycin, 0.5 μg /ml of FSH, 0.5μg /ml of LH, 10 ng/ml of the epidermal growth factor (EGF) and 0.57 mM cysteine. About 80 oocytes were cultured in 500 μl of maturation medium covered with 200 μl mineral oil for 44 hours at 38.8 °C in a humified 5% CO_2_ atmosphere in a four well culture dish (Nunc, oskide, Denmark).

### Antibodies and chemicals

Rabbit monoclonal anti-p-MAPK antibody, rabbit monoclonal anti-LC3 antibody were purchased from Cell Signaling Technology (Beverly, MA, USA). Alexa Fluor 488 goat anti-rabbit antibody and Alexa Fluor 594 goat anti-mouse antibody were purchased from Invitrogen (Carlsbad,CA). Dihydroethidium was from the Beyotime Institute of Biotechnology (Nantong, China). Annexin V-FITC/EGFP Apoptosis Detection Kits were from Vazyme Biotech Co.Ltd. (Nanjing, China). HT-2 toxin was from J&K Chemical Ltd. (Shanghai, China). Other chemicals were from Sigma Chemical Company (St. Louis, MO).

### HT-2 treatment

HT-2 was dissolved in DMSO (the original concentration was 1 mM) and then diluted with a maturation medium.

### Immunofluorescence microscopy

Oocytes in control group have reached MI stage after being cultured for 28 hours while in treat group they need to be cultured for 44 hours to reach MI stage due to the toxic effects. It was indicated by a metaphase plate without polar body or clumped condensed chromatin. The cumulus cells were removed by repeated pipetting as we need the denuded oocytes for observation. Then, the oocytes were fixed with 4% paraformaldehyde for 30 minutes and permeablized with 1% Triton X-100 at room temperature for at least 8 h. Subsequently, the oocytes were blocked with 1% BSA-supplemented phosphate-buffered saline (PBS) for 1 h, then the oocytes were stained with different primary antibodies (anti-α-tubulin-FITC at 1:400; 5 μg/ml of Phalloidin-TRITC; LC3 at 1:100) at room temperature for 1 h. After washing for three times, the oocytes were stained with an Alexa Fluor 488 goat anti-rabbit antibody (1:500) at room temperature for 1 h.

After staining, the specimens were mounted on glass slides and then examined with a Confocal Laser-scanning Microscope (Zeiss LSM 700 META, Germany).

### ROS detection

To detect intracellular ROS levels, a method to detect ROS as green fluorescent signals of DCFH diacetate (DCHFDA; Beyotime Institute of Biotechnology, China) was used. The oocytes were incubated at 37 °C for 30 min in DPBS that contained DCHFDA (1:800). After three times washes in DPBS, the samples were mounted on glass slides with DPBS and covered with cover slips. The oocytes were determined immediately with a confocal laser-scanning microscope (Zeiss LSM700 META).

### Annexin-V staining of oocytes

An Annexin-V staining kit was used for the detection of early-apoptosis (Vazyme, Nanjing, China). The oocytes were incubated at room temperature in the dark for 10 min with 100 ml of binding buffer containing 10 ml of Annexin-V-EGFP. Other steps are the same as the detection of ROS.

### Western Blot analysis

About 100 oocytes were collected in both control and treatment group and lysed in Laemmli sample buffer (SDS sample buffer and 2-Mercaptoethanol) and boiled at 100 °C for 10 min, then transferred into a refrigerator to be frozen at −20 °C until use. Proteins were separated by sodium dodecyl sulfare-polyacrylamide gel electrophoresis (SDS-PAGE) with a Criterion precast gel (Bio-Rad, Richmond, CA, USA).Then, proteins were electrophoretically transferred onto a polyvinylidene fluoride membrane (Millipore, Billerica, MA) for 1.5 h at 100 V at 4 °C. The membranes were blocked 1 h with Tris-buffered saline (TBS) containing 0.1% (w/w) Tween 20 (TBST) and 5% (w/v) nonfat dry milk powder for 1 h and then incubated with various antibodies (anti-α-tubulin antibody, anti-p-MAPK antibody) overnight at 4 °C. After washing several times in Tris-buffered saline containing 0.1% Tween 20 and 5% no fat dry milk and incubating with anti-rabbit horseradish peroxidase linked antibody for 1 h, detection of α-tubulin by enhanced chemiluminescence was done to perform equal protein loading.

### Fluorescence intensity analysis

The samples of control and treated oocytes were mounted on the same glass slide for fluorescence intensity. We used the Confocal Laser-scanning Microscope to do the immunofluorescence microscopy and the same parameters were used to normalize across the replicates. At least three replicates were used for each treatment and no less than 30 oocytes were examined for each replicate. The average fluorescence intensity per unit area within the region of interest (ROI) of immunofluorescence images was examined. The fluorescence intensity was assessed using Image J software (NIH). When calculating the fluorescence intensity, we neglected the abnormal ones (unusual oocytes with extremely strong or weak intensity). The average values of all measurements were used to determine the final average intensity for the control and treated oocytes.

### Statistical analysis

At least three replicates were used for each treatment and no less than 30 oocytes were examined for each replicate. The results are given as means ± SEMs. Statistical comparisons were made by analysis of variance (ANOVA), and differences between treatment groups were assessed by Duncan’s multiple comparisons. A p-value of <0.05 was considered statistically significant.

## Additional Information

**How to cite this article**: Zhang, Y. *et al.* Exposure to HT-2 toxin causes oxidative stress induced apoptosis/autophagy in porcine oocytes. *Sci. Rep.* **6**, 33904; doi: 10.1038/srep33904 (2016).

## Supplementary Material

Supplementary Information
