# Supplementary material for: Exposure to HT-2 toxin causes oxidative stress induced apoptosis/autophagy in porcine oocytes
Source: Sci Rep. 2016 Sep 23;6:33904. doi: 10.1038/srep33904 (PMC5034267; doi:10.1038/srep33904)

Exposure to HT-2 toxin causes oxidative stress induced apoptosis/autophagy in porcine oocytes

Yue Zhang^1^, Jun Han^1^, Cheng-Cheng Zhu^1^, Feng Tang^1^, Xiang-Shun Cui^2^, Nam-Hyung Kim^2^, Shao-Chen Sun^1^*

^1^College of Animal Science and Technology, Nanjing Agricultural University, Nanjing 210095, China.

^2^Department of Animal Sciences, Chungbuk National University, Cheongju 361-763, Korea.

**^*^Correspondence to:** Shao-Chen Sun, College of Animal Science and Technology, Nanjing Agricultural University, Nanjing, China. Tel/Fax: +86-25-84399092. E-mail: [sunsc@njau.edu.cn](mailto:sunsc@njau.edu.cn)

Supplementary figure: The original figure for the bands of p-MAPK (42/44 kDa), α-tubulin (52kDa) and the band marker for the western blot experiments.


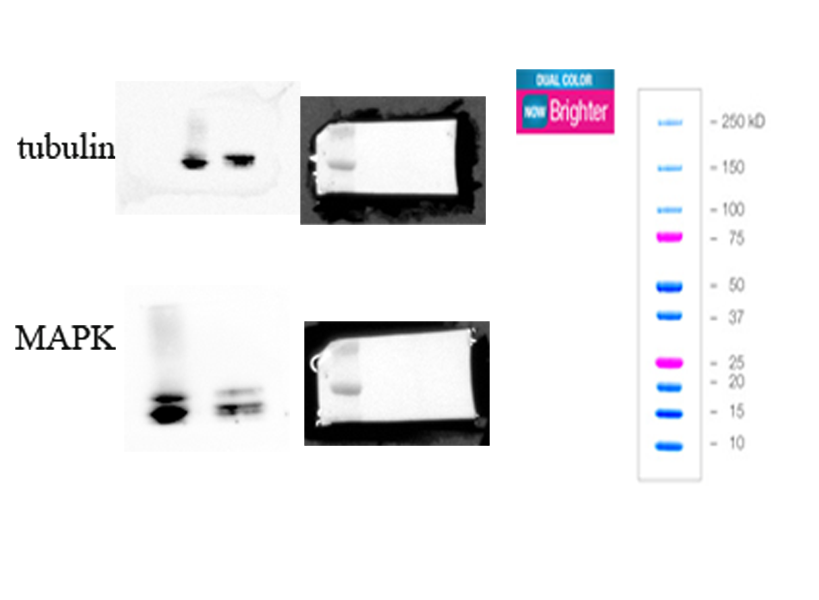

Supplement: Supplementary Information [file srep33904-s1.doc]
